# The cost-effectiveness of using vilobelimab with immunomodulators to treat severely ill mechanically ventilated patients with COVID-19: A subgroup analysis of the PANAMO study

**DOI:** 10.1093/ajhp/zxaf174

**Published:** 2025-07-10

**Authors:** Daniel C Malone, Roger Seheult, Diana Brixner, Gary Oderda, Bruce P Burnett, Joseph Biskupiak

**Affiliations:** College of Pharmacy, University of Utah, Salt Lake City, UT, USA; University of California Riverside School of Medicine, Riverside, CA; Loma Linda University School of Medicine, Lorna Linda, CA, USA; College of Pharmacy, University of Utah, Salt Lake City, UT, USA; College of Pharmacy, University of Utah, Salt Lake City, UT, USA; InflaRx Pharmaceuticals, Inc., Ann Arbor, MI, USA; College of Pharmacy, University of Utah, Salt Lake City, UT, USA

**Keywords:** cost effectiveness, COVID-19, immunomodulators, quality-adjusted life years, vilobelimab

## Abstract

**Purpose:**

The anti–C5a complement factor blocker vilobelimab (Gohibic) is authorized for emergency use by the US Food and Drug Administration for the treatment of hospitalized adults with COVID-19 when initiated within 48 hours of starting invasive mechanical ventilation or extracorporeal membrane oxygenation. In the PANAMO phase 3 trial of vilobelimab vs standard of care (SoC) (corticosteroids and antithrombotic agents), a post hoc analysis showed that prior and/or concomitant treatment with immunomodulators (tocilizumab or baricitinib) provided additional survival benefit with vilobelimab (n = 34) but not with SoC (n = 37). The point estimate for 28-day all-cause mortality for these groups was 6.3% vs 40.9% (hazard ratio, 0.13; 95% confidence interval, 0.03-0.56; *P* = 0.006). The current analysis evaluated the cost-effectiveness of vilobelimab vs SoC for the subgroup in the PANAMO study receiving immunomodulators.

**Methods:**

A short-term acute care decision tree followed by a postdischarge 2-state Markov cohort model was used to estimate quality-adjusted life years (QALYs) and the incremental cost-effectiveness ratio (ICER) of the treatment arms. The model simulated progression from severe COVID-19 to survival or death over a lifetime. Outcomes data (COVID-19 all-cause mortality) were incorporated from PANAMO. Posthospitalization mortality was based on CDC age-specific survival data. Utility values and hospital costs came from the literature, and the cost of vilobelimab was obtained from Red Book online.

**Results:**

The total cost of care was $103,414 with SoC and $132,247 with vilobelimab, with an incremental cost of $28,833. SoC provided 6.05 QALYs vs 9.71 QALYs for vilobelimab (3.65 additional QALYs). The ICER for vilobelimab compared to SoC, both with immunomodulators, was $7,892/QALY. Probabilistic sensitivity analysis demonstrated the robustness of the cost-effectiveness result, as vilobelimab + SoC was favored at a willingness-to-pay threshold of $50,000/QALY in over 98% of iterations.

**Conclusion:**

Vilobelimab therapy provides a cost-effective option to treat severely ill, mechanically ventilated patients with COVID-19 with prior and/or concomitant treatment with immunomodulators at a value well below the commonly accepted willingness-to-pay threshold of $50,000/QALY.

Key Points

- For critically ill COVID-19 patients receiving invasive mechanical ventilation who had prior and/or concomitant treatment with immunomodulators (tocilizumab or baricitinib) in addition to the standard of care, vilobelimab provides a significant additional mortality benefit at 28 and 60 days versus standard of care.
- There was no mortality benefit of IL-6 immunomodulatory agents when administered to this patient population with placebo in addition to standard of care.
- A cost-effectiveness analysis shows adding vilobelimab to standard of care with other immunomodulators for critically ill COVID-19 patients is cost-effective at a value well below the commonly accepted $50,000 willingness-to-pay threshold.

As of December 2024, the US had reported a total of approximately 112 million coronavirus disease 2019 (COVID-19) cases and about 1.22 million deaths since the beginning of the pandemic.^1^ Monthly rates of COVID-19 hospitalization have declined steadily since the beginning of the pandemic. In November 2024, the monthly rate was 7.0 hospitalizations per 100,000, whereas the rate in November 2020 was 70.4 hospitalizations per 100,000.^1^ In the early stages of the pandemic, several studies in New York City from March through April 2020 found that about 1 in 4 patients hospitalized with COVID-19 became critically ill, with approximately 80% of critically ill patients requiring mechanical ventilation.^2^ Because of changes in reporting requirements over the last 2 years, it is difficult to determine the exact number of patients with COVID-19 who were mechanically ventilated. Starting in November 2024, the US federal government again required hospitals to report hospitalizations and intensive care unit (ICU) admissions after a 7-month pause.^3^ From December 2023 to December 2024, the rate of ICU admission, which may be considered to be a surrogate for the rate of ventilation, varied between 1% and 4% of almost 105,000 ICU beds nationally each week.^4^ Acute kidney injury is common among critically ill patients with COVID-19 (occurring in up to 80% of cases), and about 15% to 30% of these patients require renal replacement therapy.^2^ A meta-analysis of studies from the US, Europe, and Asia included 10,150 patients admitted to the ICU with COVID-19 and found that, in studies with complete ICU disposition data (ie, death or discharge), the combined ICU mortality rate was 41.6%.^5^ Even though current estimates of the proportion of patients with COVID-19 receiving invasive mechanical ventilation (IMV) or extracorporeal membrane oxygenation (ECMO) are low, the risk for viral-induced sepsis or acute respiratory distress syndrome with high rates of mortality remains high in such critically ill patients, thus demonstrating an unmet need in this population.^6-8^

Vilobelimab (Gohibic), the only immunomodulator tested exclusively in a randomized, placebo-controlled, and properly powered (90%) study of mechanically ventilated patients with COVID-19, has been issued an emergency use authorization (EUA) by the Food and Drug Administration (FDA) for the treatment of COVID-19 in hospitalized adults when initiated within 48 hours of patients starting IMV or ECMO.^9^ Vilobelimab is a C5a complement factor–specific blocker that was demonstrated to lead to a significant reduction in the rate of 28-day all-cause mortality in the PANAMO phase 3 global study in adult critically ill patients with COVID-19 (N = 368) when used in addition to standard of care (SoC) (32% mortality compared to 42% with SoC alone; hazard ratio [HR], 0.67; 95% confidence interval [CI], 0.48-0.96; *P* = 0.027).^10^ A cost-effectiveness analysis (CEA) of the population in the PANAMO study^10^ has already been reported and demonstrated that, for ICU patients with COVID-19, the incremental cost-effectiveness ratio (ICER) for vilobelimab compared to SoC alone was $22,287 per quality-adjusted life year (QALY).^11^

In preclinical models, C5a induces higher interleukin (IL)–6 levels and STAT3 expression, as well as activation of signaling in the JAK-STAT pathway, suggesting an interplay between C5a- and IL-6–induced inflammatory pathways involved in severe COVID-19.^12-15^ In the PANAMO study, SoC included the use of corticosteroids (99%) and antithrombotic agents (98%). Immunomodulators (n = 71; 19.3%), including tocilizumab (n = 61) and/or baricitinib (n = 12) (2 patients received both tocilizumab and baricitinib), were allowed in the study per local guidelines for the treatment of COVID-19. Subgroup analysis of patients receiving treatment with immunomodulators in the PANAMO study found a strong potentially synergistic survival benefit when vilobelimab was given to patients with COVID-19 on IMV or ECMO along with concomitant treatment with tocilizumab or baricitinib. No benefit was seen with IL-6 immunomodulatory agents when they were administered with placebo. The 28-day all-cause mortality rate was 6.3% for vilobelimab + immunomodulatory agent + SoC vs 40.9% for placebo + immunomodulatory agent + SoC (HR, 0.13; 95% CI, 0.03-0.56; *P* = 0.006).^16^

## Objective

The objective of this analysis was to evaluate the cost-effectiveness of treatment with vilobelimab + SoC vs placebo + SoC among the subgroup of patients in the PANAMO study receiving immunomodulators as part of SoC per local guidelines during the trial.

## Methods

A previously described cost-effectiveness model was utilized^11^ and data from the immunomodulator subgroup in PANAMO^16^ were incorporated to estimate the impact of vilobelimab + SoC with immunomodulators (vilobelimab group) relative to placebo + SoC with immunomodulators (SoC group) for hospitalized patients with severe COVID-19 requiring IMV. It should be noted that the only difference from the previous CEA was the incorporation of the treatment effect in the immunomodulator subgroup and the age of patients in the immunomodulator subgroup.

As in the previous CEA, a lifetime model was constructed from a modified societal perspective that included a short-term acute care decision tree followed by a postdischarge 2-state Markov cohort model with a cycle length of 1 month and half-cycle correction. The model estimated progression from severe COVID-19 to survival or death and the receipt of renal replacement therapy. Survivors then transitioned to the Markov section of the model, where the probability of a transition from a health state of alive to dead was based on life table values from the Centers for Disease Control and Prevention.^17^

Clinical outcomes data for the vilobelimab and SoC groups were derived from the post hoc analysis of patients who also received immunomodulator therapy in the phase 3 PANAMO study and are shown in Table 1.^16^ Sixty-day all-cause mortality data were used to capture all survival data available from the trial. At day 60, 17.6% of patients in the vilobelimab group had died, compared to 48.6% of patients in the SoC group. Patients in the immunomodulator subpopulation in PANAMO were slightly older than those in the total PANAMO population (58.6 vs 56.3 years, respectively); therefore, in the base case, patients entered the model at an age of 58.6 years. Because both groups received tocilizumab and/or baricitinib, the cost of immunomodulator medications was not included in the model. All other model inputs were the same as reported in the previous CEA.

**Table 1.** Clinical Feature and Cost Estimates

	Base case	Range	Distribution	Source
**Clinical characteristics^a^**				
60-day survival for vilobelimab	0.82	0.65 to 0.93	Beta	Shorr et al^16^
60-day survival for SoC	0.51	0.34 to 0.66	Beta	Shorr et al^16^
Age, mean, years	58.6	22 to 79	Normal	Shorr et al^16^
Renal replacement therapy for vilobelimab	0.096	0.086 to 0.106	Beta	Vlaar et al^10^
Renal replacement therapy for SoC	0.157	0.141 to 0.173	Beta	Vlaar et al^10^
Duration of renal replacement therapy for vilobelimab, days	6.8	7.8 to 8.8	Gamma	Vlaar et al^10^
Duration of renal replacement therapy for SoC, days	9.4	8.1 to 10.7	Gamma	Vlaar et al^10^
**Costs**				
Renal replacement therapy per day	$2,000	$1,800 to $2,200	NA	Tseng et al^18^
Vilobelimab per administration	$6,350	$5,715 to $6,985	NA	Red Book^18^
Intensive care treatment of COVID-19	$100,461	$40,218 to $100,461	NA	Kaiser Family Foundation report^19^

Abbreviations: COVID-19, coronavirus disease 2019; NA, not applicable.

^a^Data without units shown are probabilities.

As this was a modeling exercise, no institutional review board approval was necessary (and thus no number was assigned) because this did not fall under the board’s classification of human subjects research. Only data from clinical trial results that have been published previously were used. The research was conducted according to the principles of the seventh revision of the Declaration of Helsinki.^21^

## Results

The base case analysis describes the cost-effectiveness of treating hospitalized patients with severe COVID-19 requiring IMV. Treatment with vilobelimab + SoC with immunomodulators (vilobelimab group) resulted in 9.71 QALYs, compared to 6.05 QALYs for placebo + SoC with immunomodulators (SoC group). The associated costs were $132,247 for the vilobelimab group and $103,414 for the SoC group. The ICER for the vilobelimab group compared to the SoC group was $7,892/QALY. In addition, treatment with vilobelimab resulted in a gain of 18.35 LYs and 11.03 equal value of life years gained (evLYG) compared to 11.44 LYs and 6.88 evLYG with SoC.

**Figure 1.**
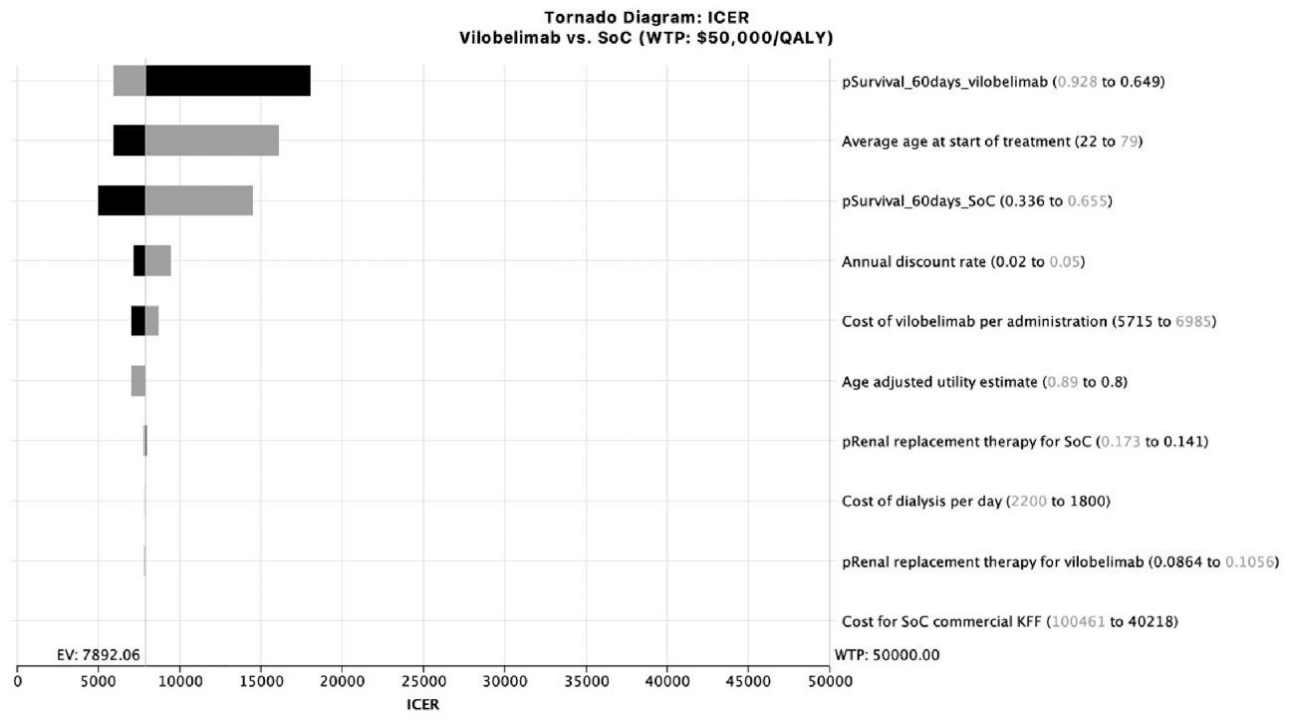
Incremental cost-effectiveness ratio (ICER) tornado diagram comparing vilobelimab (Vilo) vs standard of care (SoC). Costs for SoC are from a Kaiser Family Foundation (KFF) report.^20^ EV indicates expected value; WTP, willingness to pay.

**Figure 2.**
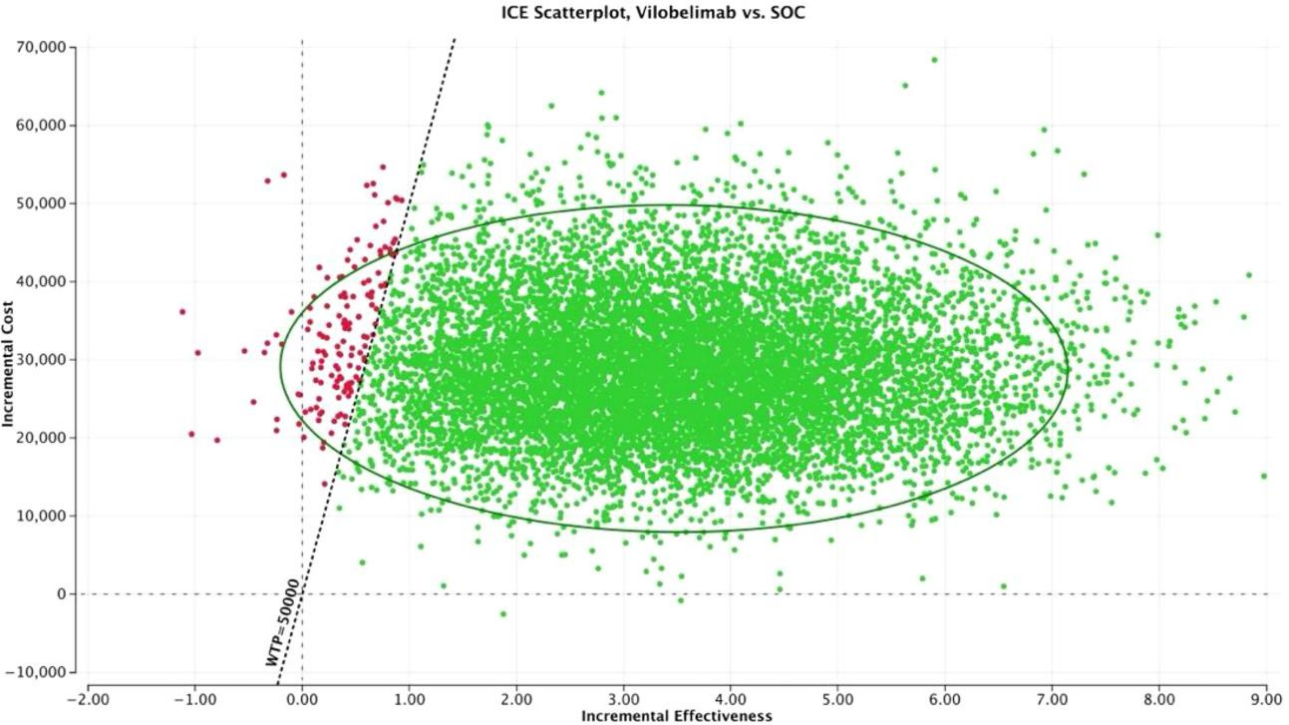
Incremental cost-effectiveness ratio (ICER) scatterplot. SoC indicates standard of care; WTP, willingness to pay.

**Figure 3.**
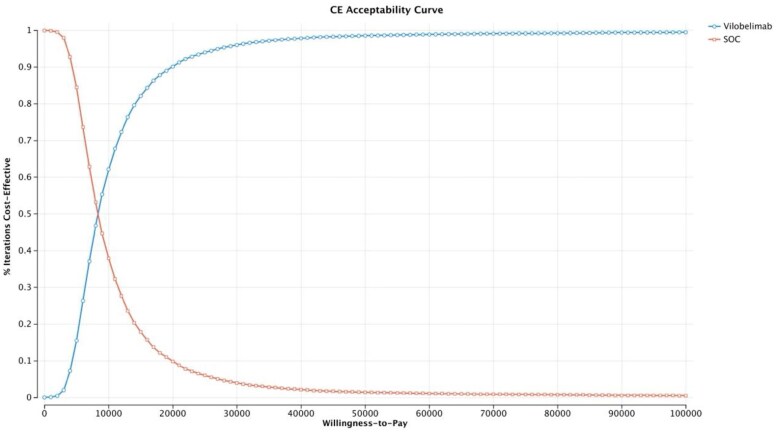
Cost-effectiveness (CE) acceptability curve.

One-way sensitivity analysis (Figure 1) found that the parameters with the greatest impact on the ICER were survival rate (vilobelimab: baseline value, 82%; range, 65% to 93%; SoC: baseline value, 51%; range, 34% to 66%) and age (baseline value, 58.6 years; range, 22 to 79 years) (Table 2). The ICER of the vilobelimab group compared to the SoC group increased with decreasing survival probability of vilobelimab, increasing age, and increasing survival probability of SoC (Table 2 and Table 3). At no point did the ICER of the vilobelimab group cross the willingness-to-pay (WTP) threshold of $50,000/QALY. In the worst-case scenario (ie, the lower bound of the survival probability with vilobelimab), the ICER was $18,058/QALY. In the best-case scenario (ie, the lower bound of the survival probability with SoC), the ICER was $5,018/QALY. Probabilistic sensitivity analysis (PSA) demonstrated that the cost-effectiveness results were highly robust to uncertainty. The PSA was run with 10,000 draws to produce stable results. Greater than 98% of the draws had an ICER per QALY gained below the $50,000 WTP threshold (Figure 2). The cost-effectiveness acceptability curve also demonstrated that greater than 98% of the draws had an ICER per QALY gained below the $50,000 WTP threshold (Figure 3).

**Table 2.** Results of One-Way Sensitivity Analysis (Top 3 Variables)

Variable	Variable values			Impact on ICER	ICER	
	Low	Base	High		Low	High
Survival probability day 60, vilobelimab	0.65	0.82	0.93	Decrease	$5,902	$18,058
Age, years	22	58.6	79	Increase	$5,929	$16,144
Survival probability day 60, SoC	0.34	0.51	0.66	Increase	$5,018	$14,518

Abbreviations: ICER, incremental cost-effectiveness ratio; SoC, standard of care.

**Table 3.** Results of Base Case Sensitivity Analysis

Strategy	Cost	Incremental cost	QALYs	Incremental effect on QALYs	ICER
SoC	$103,414	Reference	6.05	Reference	Reference
Vilobelimab	$132,247	$28,833	9.71	3.65	$7,892

Abbreviations: ICER, incremental cost-effectiveness ratio; QALYs, quality-adjusted life years; SoC, standard of care.

## Discussion

Using data from the immunomodulator subgroup population of the phase 3 PANAMO study, the CEA of treatment with vilobelimab + SoC with immunomodulators compared to placebo + SoC with immunomodulators demonstrated the value of treatment with vilobelimab, with the ICER/QALY well below the commonly accepted WTP threshold in the US of $50,000/QALY. Given that the costs were the same as in the previous CEA and the only change was the improvement in effectiveness, this result was entirely predictable. It should be noted that, if the costs of immunomodulator therapy had been included in this analysis, the total cost for both groups would be slightly higher, but the difference in costs would be the same and the ICER would also be the same.

Both baricitinib and tocilizumab were shown to improve 28-day survival in patients admitted to the hospital with COVID-19 requiring oxygenation (low-flow cannula to mechanical ventilation) in a randomized, controlled, open-label platform study (RECOVERY).^22,23^ In RECOVERY, 514 of 4,148 patients (12%) allocated to baricitinib vs 546 of 4,008 patients (14%) allocated to usual care died within 28 days (age-adjusted rate ratio of 0.87; 95% CI, 0.77-0.99; *P* = 0.028). Furthermore, 621 (31%) of the 2,022 patients allocated to tocilizumab and 729 (35%) of the 2,094 patients allocated to usual care died with 28 days (rate ratio of 0.85; 95% CI, 0.76-0.94; *P* = 0.028). While these findings are encouraging, in patients receiving IMV at randomization, no survival benefit was observed with either baricitinib or tocilizumab. The age-adjusted rate ratio of death was 0.93 (95% CI, 0.61-1.44) for baricitinib and 0.93 (95% CI, 0.74-1.18) for tocilizumab. Tocilizumab has been approved by FDA with evidence that utilization improves outcomes in a broader population of hospitalized patients with COVID-19 in need of supplemental oxygen, while baricitinib has an EUA. Vilobelimab is, to date, the only host-response-directed therapy demonstrated to result in a survival benefit when administered after onset of IMV.

The potential additional impact on survival of immunomodulator therapy along with vilobelimab in the subanalysis of the PANAMO trial warrants further investigation in larger populations to better understand the biological cause for this potential additional survival effect.

### Limitations

The model utilized a lifetime perspective, although mortality data were available for only 28 and 60 days. Patients who were discharged from the hospital during the study were assumed to have a similar life expectancy based on their age at discharge, although disutility values were applied for the first 4 years because all patients required mechanical ventilation (Table 4). Hospital costs were assumed to be the same in both groups except for the cost of vilobelimab and renal replacement therapy. The model also assumes that long-term life expectancy is not affected by receiving IMV due to COVID-19.

**Table 4.** US Age-Specific Utility and Disutility Values for Mechanical Ventilation and Postdischarge Disutility for Patients Requiring Mechanical Ventilation Over 4 Years^24^

	Base case	SD	Distribution
US age-specific utility			
40-49 years	0.870	0.002	Beta
50-59 years	0.840	0.003	Beta
60-69 years	0.820	0.003	Beta
70-79 years	0.790	0.004	Beta
>80 years	0.740	0.006	Beta
Mechanical ventilation during hospitalization disutility	0.560	0.300	Beta
Postdischarge disutility for patients requiring mechanical ventilation			
Year 1	0.130	0.013	Beta
Year 2	0.067	0.007	Beta
Year 3	0.062	0.006	Beta
Year 4	0.026	0.001	Beta

Additional limitations were due to the data used to populate the model, which were supported by a clinical study that was global and approximated a US population but was conducted outside the US. The model inputs were also subject to the restrictions of a clinical study. In addition, this study utilized data from a post hoc analysis of a small subgroup that received the immunomodulators baricitinib and/or tocilizumab. Despite utilizing data from a post hoc analysis, a power analysis was conducted of this subgroup and showed post hoc power of 95%. It should also be noted that many patients received prior or concomitant treatment with tocilizumab (n = 61) while fewer patients received treatment with baricitinib (n = 12). These limitations can be addressed as real-world data on the utilization, outcomes, and costs of vilobelimab become available following FDA approval.

## Conclusions and relevance

For the subgroup of severely ill, mechanically ventilated patients with COVID-19 with prior and/or concomitant immunomodulator therapy, treatment with vilobelimab + SoC can increase the patient’s odds of survival. This study showed that treatment with vilobelimab + SoC with prior and/or concomitant immunomodulator use is also a cost-effective option, with an ICER of $7,892/QALY, which is well below what is commonly considered the acceptable WTP threshold of $50,000/QALY. Considering these results, providers may be encouraged to consider adding vilobelimab to treatment regimens for patients with COVID-19 receiving IMV.
